# Statistical Approach to Spectrogram Analysis for Radio-Frequency Interference Detection and Mitigation in an L-Band Microwave Radiometer

**DOI:** 10.3390/s19020306

**Published:** 2019-01-14

**Authors:** Myeonggeun Oh, Yong-Hoon Kim

**Affiliations:** School of Mechatronics, Gwangju Institute of Science and Technology, 123 Cheomdangwagi-ro, Buk-gu, Gwangju 61005, Korea; mgoh1815@gmail.com

**Keywords:** microwave radiometer, kurtosis, skewness, spectrogram, radio-frequency interference (RFI)

## Abstract

For the elimination of radio-frequency interference (RFI) in a passive microwave radiometer, the threshold level is generally calculated from the mean value and standard deviation. However, a serious problem that can arise is an error in the retrieved brightness temperature from a higher threshold level owing to the presence of RFI. In this paper, we propose a method to detect and mitigate RFI contamination using the threshold level from statistical criteria based on a spectrogram technique. Mean and skewness spectrograms are created from a brightness temperature spectrogram by shifting the 2-D window to discriminate the form of the symmetric distribution as a natural thermal emission signal. From the remaining bins of the mean spectrogram eliminated by RFI-flagged bins in the skewness spectrogram for data captured at 0.1-s intervals, two distribution sides are identically created from the left side of the distribution by changing the standard position of the distribution. Simultaneously, kurtosis calculations from these bins for each symmetric distribution are repeatedly performed to determine the retrieved brightness temperature corresponding to the closest kurtosis value of three. The performance is evaluated using experimental data, and the maximum error and root-mean-square error (RMSE) in the retrieved brightness temperature are served to be less than approximately 3 K and 1.7 K, respectively, from a window with a size of 100 × 100 time–frequency bins according to the RFI levels and cases.

## 1. Introduction

The presence of radio-frequency interference (RFI) is a significant issue in radiometric measurements, potentially caused by any type of electromagnetic emission, such as those from communication and navigation systems. Even if microwave radiometry is utilized for measuring sea salinity and soil moisture within the protected bandwidth range of 1400 to 1427 MHz, RFI can still degrade radiometric measurements owing to leakages from adjacent bands [1,2]. Specifically, the effect on the RFI adjacent to the land is critical for the ground-based microwave radiometer for remote sensing outdoor application when compared with the measurements from the open ocean using airborne and satellite [3,4,5,6]. Moreover, RFI at a higher frequency such as at the C- [7], X- [5,6,7,8], and Ku-bands [9] can arise in the microwave radiometry field. Recently, ground-based microwave radiometers have been used for Internet of Things (IoT) applications [10] and for effective detection and mitigation of RFI contamination. Several methods have been developed to detect and mitigate the presence of RFI. A powerful means of RFI detection and mitigation is the use of the kurtosis technique [11,12,13,14]. The kurtosis is measure of the “peakedness” and “tailedness” of the probability distribution and can be computed as Refs. [15,16,17,18].

(1) $$Kurt=\frac{\mu_{4}}{\sigma^{4}}$$

where σ and $\mu_{4}$ are the standard deviation and the fourth central moment, respectively. The kurtosis value is equal to three if the received signal is RFI-free data; however, kurtosis uncertainty arises owing to a finite number of samples [2]. Moreover, the kurtosis value can still be three for signal types such as the chirp and sinusoidal RFI signals. In other methodologies, RFI can be eliminated using time and frequency-domain techniques [19,20,21]. However, a method based on only one technique can not sufficiently remove contaminated RFI signals [22,23,24]. Recently, a multi-domain technique that combines a brightness temperature spectrogram, kurtosis spectra, and a time-domain plot was introduced for the Soil Moisture Active Passive (SMAP) mission [22]. From experimental results, the presence of various types of RFI signals was more clearly detected using the spectrogram technique than by using any other techniques. Specifically, chirp RFI signals were not observed in the time domain and kurtosis spectra. To eliminate RFI contamination in the brightness temperature spectrogram, the threshold level of the brightness temperature spectrogram was calculated as *m + βσ* for the lowest 90% of the corresponding bins, where *m* is the mean value, *β* is the threshold multiplier, and *σ* is the standard deviation. However, a higher threshold level can be determined if the presence of RFI exceeds the highest 10% in those bins. Thus, the selection of a constant percentage for those bins leads to an error in the retrieved brightness temperature. Although the RFI is eliminated via the increased threshold level, significant error will arise. As an alternative to the spectrogram technique, an image processing technique was employed using a smoothing algorithm [25]. In these results, various RFI signal types must be considered to determine the threshold multiplier for outdoor experiments beforehand, as threshold multipliers for the optimum threshold depend on RFI signal types with different values.

The aim of this paper is to demonstrate RFI detection capabilities and to present a mitigation algorithm for the purpose of minimizing potential errors originating from the sample intensity level using the threshold level from statistical criteria based on a spectrogram technique. A discussion of the proposed method follows in Section 2. This experiment was performed using a microwave radiometer system operating on the L-band. The instrument and test setup are described in Section 3. The experimental results and analysis are presented in Section 4, and the conclusion is presented in Section 5.

## 2. Proposed RFI Detection and Mitigation Algorithm

**Figure 1:**
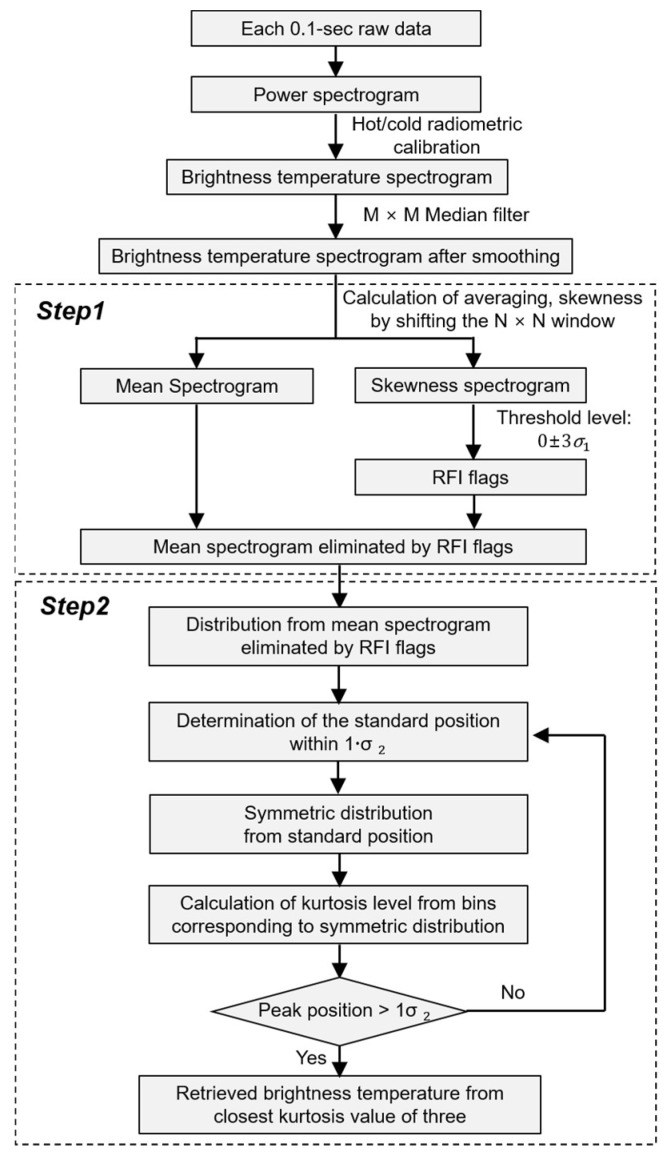
Flowchart of the proposed process.

With existing techniques, the threshold level [22] is calculated as the mean value plus three times the standard deviation of measured quantities; however, this type of threshold level can lead to significant errors stemming from increases in the mean value and standard deviation which occur when increasing the RFI level and number of RFI samples for each process data. Although RFI contamination can be eliminated by increasing the value of the threshold level, the error in the retrieved brightness temperature becomes more serious when doing so. In order to solve this problem, the RFI contamination is firstly removed from the threshold level by using the skewness calculation in spectrogram technique, which is superior when used to observe various types of RFI signals compared with other techniques [22]. Here, the RFI contamination is not perfectly removed due to the higher standard deviation for threshold level and skewness uncertainty. From the Gaussian distribution of the remaining data, higher RFI signals adjacent to the average of the Gaussian distribution may be not unremoved. Thus, the kurtosis level can be calculated from the corresponding bins by shifting any point in this Gaussian distribution, and the retrieved brightness temperature can be obtained from the kurtosis curve. This can achieve a more accurate brightness temperature. These procedures for RFI detection and mitigation are shown in Figure 1. The power spectrogram can be obtained from a data stream at the digital back end, and subsequently the radiometric calibration is performed for the brightness temperature spectrogram. Each brightness temperature spectrogram is smoothed using a median filter with a size of M × M time and frequency bins [22]. The presence of RFI can be detected using the threshold level from the intensity of the bins in the brightness temperature spectrogram; however, this method incurs a considerable amount of error according to the RFI level and the number of selected bins in the brightness temperature spectrogram. To determine the retrieved brightness temperature, the subsequent steps are described as follows.

### 2.1. Step 1: Statistical Thresholding Using Skewness

**Figure 2:**
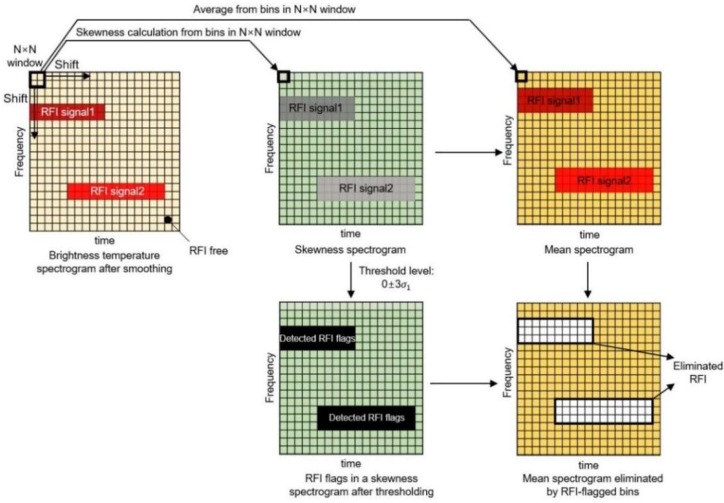
Process of obtaining the spectrograms of RFI-contaminated signals corresponding to each procedure in Step 1.

The distribution of all bins in the brightness temperature spectrogram has the form of a Gaussian distribution. The received signals, comprised of the natural thermal emission signal and the RFI contamination, show that the mass of the distribution is concentrated on the right tail because the RFI level is higher than the natural thermal emission signal. Thus, the presence of RFI can initially be detected from the asymmetry of the distribution by means of a skewness calculation. Skewness refers to asymmetry in a statistical distribution, and the skewness value can be positive, negative, or undefined. The skewness, $\gamma_{1}$, can be defined as Ref. [26]

(2) $$\gamma_{1}=\frac{\mu_{3}}{\sigma^{3}},$$

where *σ* and $\mu_{3}$ are the standard deviation and the third central moment, respectively. The calculated skewness value is equal to zero if the received signal has a symmetric distribution. From the shift of the 2-D window with a size of N × N time–frequency bins in the brightness temperature spectrogram at a constant size, the skewness levels from the bins in each window are computed to determine the asymmetry of the distribution, as shown in Figure 2. In this work, 2-D rectangular windows are used to reduce the error in the skewness on the side of the brightness temperature spectrogram. A small window can increase the uncertainty of the skewness, whereas a large window causes many bins in the skewness spectrogram to disappear considering the RFI contamination. The threshold level in the skewness spectrogram was computed as zero plus three times the standard deviation, that is, $3\sigma_{1}$, where $\sigma_{1}$ is the standard deviation of all bins in the skewness spectrogram. Using a value of zero, rather than the mean value from the constant percentage of bins in the brightness temperature spectrogram, can serve as an accurate reference [22]. Subsequently, RFI-flagged bins in the skewness spectrogram are reflected in the mean spectrogram as shown in Figure 2.

### 2.2. Step 2: Process of Obtaining the Retrieved Brightness Temperature Using Kurtosis

**Figure 3:**
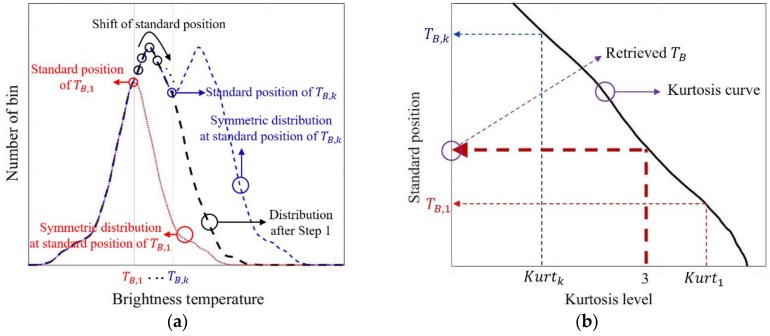
(**a**) Distribution from the remaining bins (black line) in Figure 2, and the examples of two symmetric distributions from the standard position of $T_{B,1}$ and $T_{B,k}$ (red and blue lines, respectively); (**b**) standard position of symmetric distribution versus kurtosis level.

From the remaining bins of the mean spectrogram, reflected by RFI-flagged bins in the skewness spectrogram in Figure 2, the distribution can be represented as the black line in Figure 3a. However, it might have the form of a non-Gaussian distribution owing to the unremoved RFI bins of the skewness spectrogram. As the bins corresponding to the left tail of the distribution (black line in Figure 3a) are relatively less affected by the RFI, two identical sides of distribution (i.e., symmetric distribution) can be created from the left tail of the distribution on the basis of specific points on the distribution (i.e., standard positions). The examples of two cases of symmetric distributions corresponding to the standard positions of $T_{B,1}$ and $T_{B,k}$, where $T_{B}$ is the brightness temperature and subscript “*k*” is the number of kurtosis calculation instances, are shown as red and blue lines in Figure 3a. In other words, the symmetric distributions have the same values of mean, median, and mode. Subsequently, two cases of kurtosis values in Figure 3b can be obtained as ${Kurt}_{1}$ and ${Kurt}_{k}$ for standard position of $T_{B,1}$ and $T_{B,k}$, respectively. In the same manner, the kurtosis curve as a solid line (black line) in Figure 3b can be obtained from the repeated kurtosis computation from the corresponding bins of each symmetric distribution by changing the standard position, $T_{B,1}\sim T_{B,k}$ on the distribution (black line in Figure 3a) with an interval of 0.1 K within $1\cdot \sigma_{2}$, where $\sigma_{2}$ is the standard deviation of the mean from the remaining bins in Figure 2 (i.e., 68% of data values). Here, the standard position in Figure 3b can be considered as the brightness temperature, and the retrieved brightness temperature over 0.1-s data can be obtained from the closest kurtosis value of three in Figure 3b.

## 3. L-Band Microwave and Experimental Setup

### 3.1. L-Band Radiometer Description

**Figure 4:**
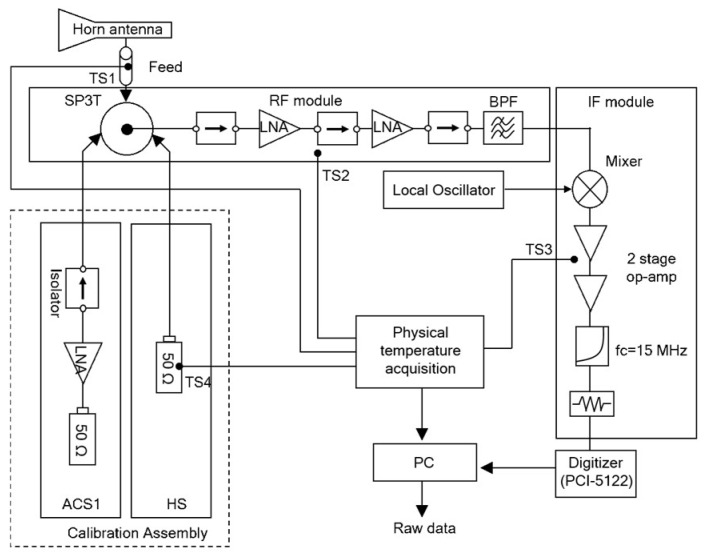
Block diagram of the developed L-band radiometer.

**Figure 5:**
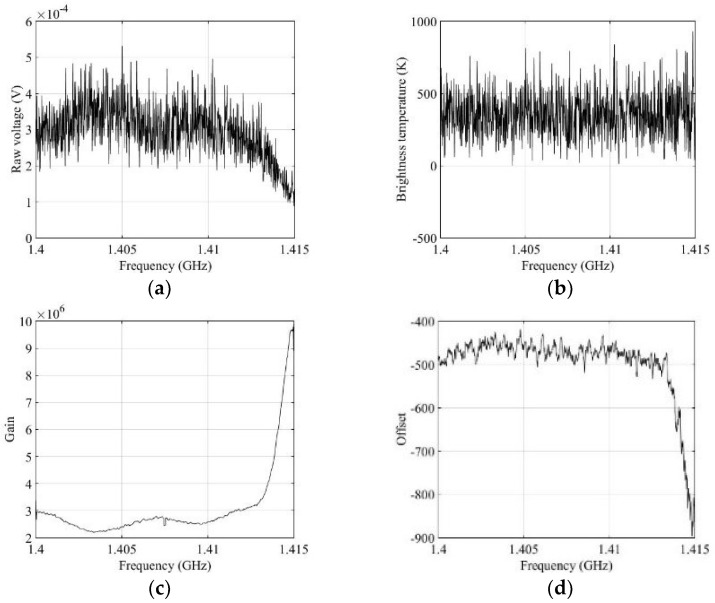
Radiometer calibration: (**a**) raw voltage data over a 0.1-s data; (**b**) brightness temperatures after radiometer calibration; (**c**) gain values; (**d**) offset values.

Figure 4 shows a block diagram of the developed L-band radiometer as a heterodyne receiver. The analog front end downconverts the 15 MHz bandwidth from 1.4 to 1.415 GHz, and samples are obtained from the digital back end at 32 MSPS with 14 bits. The radiometer receiver can be operated either as a total power radiometer in the measurement mode, or in the calibration mode [27]. In the calibration mode, two-point calibration was performed using active cold sources (ACS) and a matched load (ML) as different known noise temperatures [28,29]. The power spectrogram in the measurement mode was computed from each 0.1-s data of 3.2 Msamples, and Hanning window size of 40,000 samples with overlapping of 6.25%. Thus, a power spectrogram with a size of 1265 × 1025 for the time and frequency bins is created with a resolution of 0.1 s/1265 ≈ 79 μs and 15 MHz/1025 ≈ 14.6 kHz. For RFI-free data, the distribution of all bins in the power spectrogram might not have the form of a Gaussian distribution because the magnitude of the frequency response corresponding 15 MHz bandwidth is not uniform (flat), due to the flatness of the analog band-pass filter and the low-pass filter in the L-band radiometer. That would require a uniform magnitude of response up to the bandwidth. Un-uniformed magnitude of frequency response can be solved from the radiometric calibration by the spectrograms of an active cold source and matched load as different known temperature sources as shown in Figure 5 [22].

The value of each bin in the brightness temperature spectrogram, $T_{B}$, can be calculated as in an earlier work [22]:

$$G=\left(T_{ML}-T_{ACS}\right)/\left(V_{ML}-V_{ACS}\right),$$

$$O=T_{ML}-V_{ML}\cdot G,$$

(3) $$T_{b,i}=G\times V_{i}+O,$$

where $T_{ML}$ and $T_{ACS}$ are, respectively, the known hot and cold temperatures of the reference sources, $V_{ML}$ and $V_{ACS}$ are the corresponding mean voltages of the power spectrogram for the corresponding frequency bins, $V_{i}$ is the voltage of each bin in the power spectrogram, and G and O are the gain and offset values from the radiometer calibration, respectively. Smoothing is performed using a median filter with a size of 8 × 8 in the calibrated spectrogram. The recorded data from the digital back end in each 1.5 s data file is divided into the measurement mode from the antenna (~1 s) and the calibration mode (~0.5 s). The temperature sensors were attached to critical radiometer components for temperature drift compensation. These were a standard gain horn antenna feed, an RF module, and an IF module for the calibration source [30]. The detailed instrument specifications of the radiometer are listed in Table 1.

### 3.2. Experimental Setup

**Figure 6:**
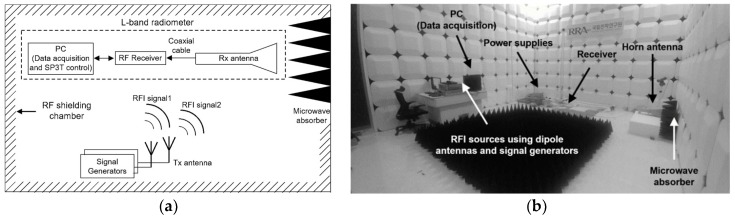
(**a**) Experimental setup; (**b**) photograph of the RFI test.

Figure 6 shows the experimental setup in the RF shielding chamber used. The ambient temperature in the RF shielding room was maintained at 296 K with a variation of 0.2 K using the thermal management controller. A pyramid-type microwave absorber was positioned in front of the horn antenna of the microwave radiometer for measured brightness temperature of 296 K, considering the emissivity of the sensed scenario equal to 1. For the true value of the brightness temperature, the generated thermal noise signal (i.e., RFI-free data) was received when the signal generators were turned off. For the performance verification of the proposed algorithm, arbitrary RFI signals were generated using two signal generators and dipole antennas. The thermal noise signal of the microwave absorber and the generated RFI signals were simultaneously obtained when the signal generators were turned on. Thus, the errors of the retrieved brightness temperature could be obtained from the difference between cases where signal generators were turned on and off. These were applied to the proposed RFI detection and mitigation algorithm, and the experiments were performed according to the RFI types and levels from the signal generators.

## 4. Experimental Results and Analysis

### 4.1. Experimental Results of Steps

**Figure 7:**
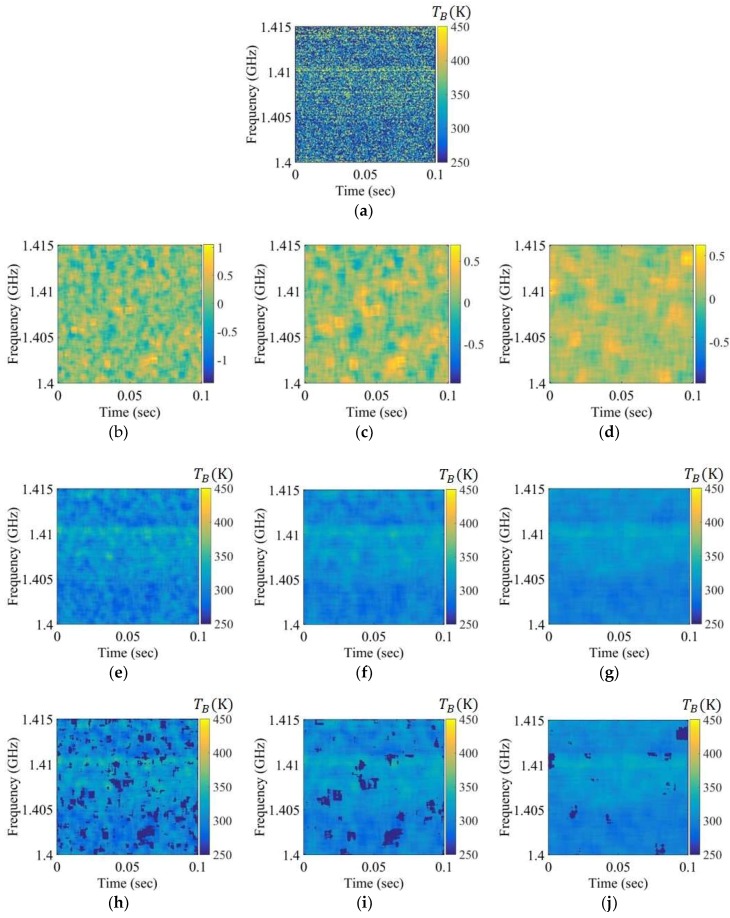
Spectrograms applied to the chirp RFI signal to a level below approximately 10 K over the entire 1.407 to 1.411 GHz band: (**a**) brightness temperature spectrogram after smoothing; (**b**–**d**) skewness spectrograms obtained using the window sizes of 50 × 50, 75 × 75, and 100 × 100, respectively; (**e**–**g**) mean spectrograms obtained using the window sizes of 50 × 50, 75 × 75, and 100 × 100, respectively; (**h**–**j**) mean spectrograms after Step 1 obtained using the window sizes of 50 × 50, 75 × 75, and 100 × 100, respectively.

**Figure 8:**
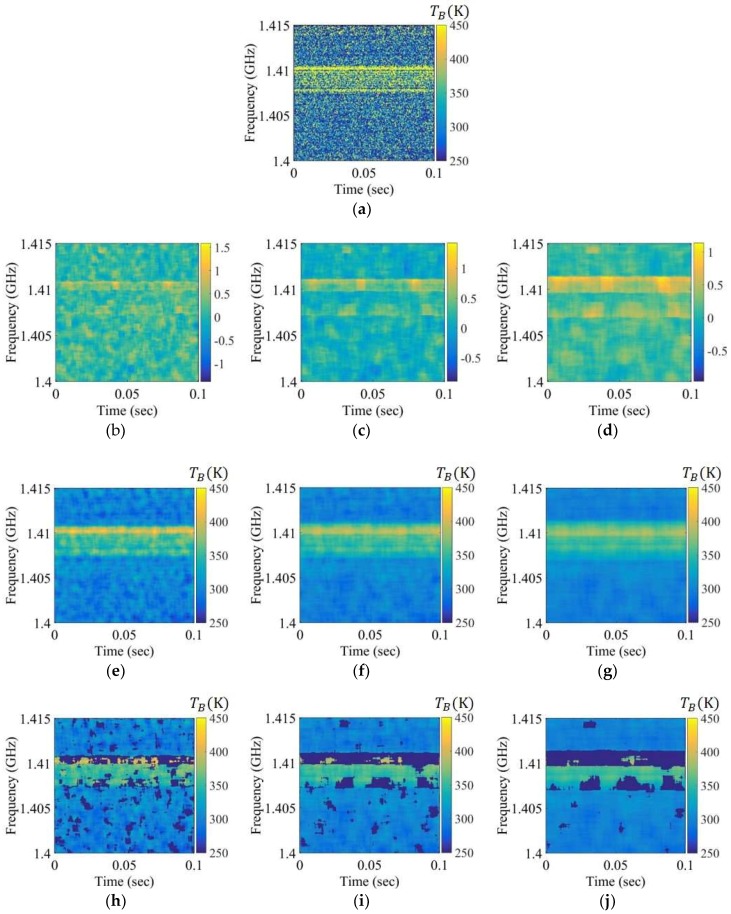
Spectrograms applied to the chirp RFI signal to a level below approximately 50 K over the entire 1.407 to 1.411 GHz band: (**a**) brightness temperature spectrogram after smoothing; (**b**–**d**) skewness spectrograms obtained using the window sizes of 50 × 50, 75 × 75, and 100 × 100, respectively; (**e**–**g**) mean spectrograms obtained using the window sizes of 50 × 50, 75 × 75, and 100 × 100, respectively; (**h**–**j**) mean spectrograms after Step 1 obtained using the window sizes of 50 × 50, 75 × 75, and 100 × 100, respectively.

**Figure 9:**
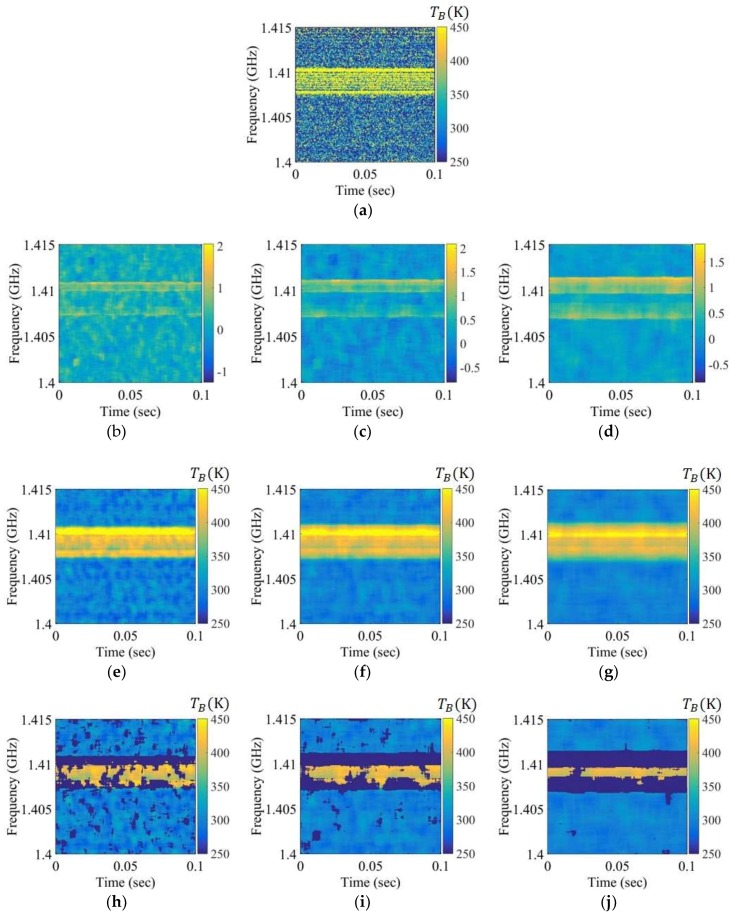
Spectrograms applied to the chirp RFI signal to a level below approximately 100 K over the entire 1.407 to 1.411 GHz band: (**a**) brightness temperature spectrogram after smoothing; (**b**–**d**) skewness spectrograms obtained using the window sizes of 50 × 50, 75 × 75, and 100 × 100, respectively; (**e**–**g**) mean spectrograms obtained using the window sizes of 50 × 50, 75 × 75, and 100 × 100, respectively; (**h**–**j**) mean spectrograms after Step 1 obtained using the window sizes of 50 × 50, 75 × 75, and 100 × 100, respectively.

**Figure 10:**
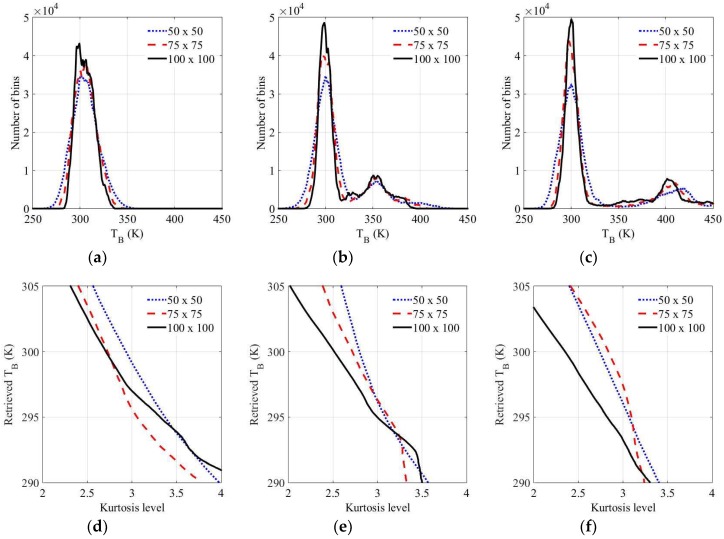
Processing results in Step 2: (**a**–**c**) distribution from the bins of the mean spectrogram eliminated by RFI-flagged bins in the skewness spectrogram when the RFI levels are 10, 50, and 100 K, respectively; (**d**–**f**) retrieved brightness temperature versus the kurtosis level when the RFI levels are 10, 50, and 100 K, respectively.

For comparison, Figure 7, Figure 8 and Figure 9 depict the experimental results for RFI chirp signals of approximately 10, 50, and 100 K level in Step 1. In the case of a weak RFI signal of the level approximately 10 K in Figure 7, it is difficult to discriminate between the natural thermal emission signal and the RFI signal in the brightness temperature spectrogram and the skewness spectrogram, because the skewness value calculated from the bins of the RFI signals and the thermal emission signals is less than the uncertainty of the skewness value calculated from the thermal emission signal. However, this signal appears in the mean spectrogram with a blurring effect. The RFI flags of the mean spectrogram become larger than those of the brightness temperature when the window size is increased. The relatively strong RFI signals of levels approximately 50 K and 100 K are detected in the spectrograms in Figure 8 and Figure 9; however, the skewness spectrogram does not eliminate the high level of RFI in the form of a Gaussian distribution. The boundaries of the RFI signal and the natural thermal emission signal in the brightness temperature spectrogram can be reliably eliminated; however, the distribution will remain symmetric if the window is covered by a wide band RFI signal. This is not an issue here because the high level of the RFI signal is separated from the natural thermal emission signal via the distribution in the examples of 350 K and 400 K in Figure 10b,c, respectively. Thus, a high level of RFI signal (e.g., RFI levels of 350 K and 400 K) can be ignored from the symmetric distributions realized by shifting the standard position of the distribution in Step 2. Figure 10d–f display the retrieved brightness temperature corresponding to each kurtosis level. According to the results, the kurtosis levels gradually decrease when the standard position of the distribution is increased. The retrieved brightness temperature can be selected from the closest value of three.

### 4.2. Error Analysis and Comparison between the Privious Method and Proposed Method

**Figure 11:**
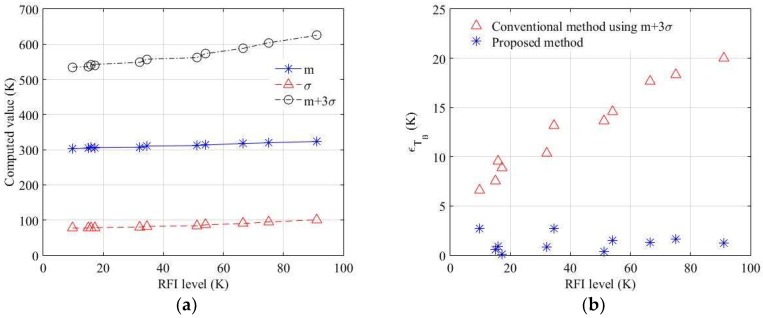
(**a**) Computed values from the bins in the brightness temperature spectrogram versus the level of chirp RFI signal occupied by the entire 1.407 to 1.411 GHz band; (**b**) error comparison of the conventional and proposed methods.

Figure 7, Figure 8, Figure 9 and Figure 10 show the experimental results obtained using the chirp RFI signal. Similarly, ten RFI signals and three window sizes were tested. The chirp RFI signal occupies the entire 1.407 to 1.411 GHz band, and the AM signal is a sinusoidal signal at 1.4045 GHz, modulated by a pulse train with a pulse width of 2 µs and pulse repetition frequency of 360 Hz with a 1% duty cycle of the pulse train. The pulsed RFI signal has a pulse period of 24 µs and a pulse width of 8 µs. The fractional bandwidth of the chirp signal is 0.214%, and the fractional bandwidths of the AM signal, continuous signal, and pulsed signal are 0.014%. The maximum errors and root-mean-square error (RMSE) of the retrieved brightness temperature are summarized in Table 2. The results in Table 2 are presented for RFI cases and levels with the same properties, such as duty cycle and period. The best performance is obtained with a window size of 100 × 100, and the maximum error of the retrieved brightness temperature, $\varepsilon_{T_{B}}$ and RMSE as tested are less than approximately 3 K and 1.7 K, respectively. A larger window size shows that the error of the retrieved brightness temperature can be reduced. In the proposed method, the error of the retrieved brightness temperature can be relatively minimized for the wide-band RFI signal. The threshold levels of the previous method are calculated as the mean value plus a few times the standard deviation from the selection of a constant percentage for the data sample [2,21,22]. However, a higher threshold level can be determined if the presence of RFI covers the wide-band and long time period. Figure 11a shows the change in the conventional threshold level using *m* + *βσ* according to the RFI level. Owing to the higher mean value and the standard deviation with the increase in the RFI level in the experimental environment of 296 K, the RFI can be properly unremoved owing to the higher bias. Figure 11b shows that, in the conventional method, the error of the brightness temperature increases from the increased RFI level. Moreover, the selection of a constant percentage for these samples leads to erroneous results for the retrieved brightness temperature. This error will be proportionally increased when the RFI is spread over a wider range. However, the proposed method can solve the problem of bias from zero in the skewness spectrogram instead of the mean value, and an appropriate value of the retrieved brightness temperature can be determined using kurtosis. It is shown that the error of RFI can be relatively reduced in a wide-band and over a long period as much as possible. 

The RFI contamination may not be completely removed due to the higher standard deviation for the threshold level in the skewness spectrogram when increasing the signal bandwidth in Step 1. Here, there is a possibility of error due to the uncertainty of kurtosis from a relatively small number of bins of the mean spectrogram eliminated by RFI-flagged bins in the skewness spectrogram [31]. The error in estimating the kurtosis from a finite number of samples, *N*, is given by

(4) $$\mathrm{NE}\Delta R=\sqrt{\frac{24}{N}}.$$

If the RFI contamination covers the entire spectrogram, it is very difficult to determine the retrieved brightness temperature because the RFI contamination in the mean spectrogram and skewness spectrogram will be entirely covered.

### 4.3. The Performance Comparison of the Skewness and Kurtosis in Step 1

**Figure 12:**
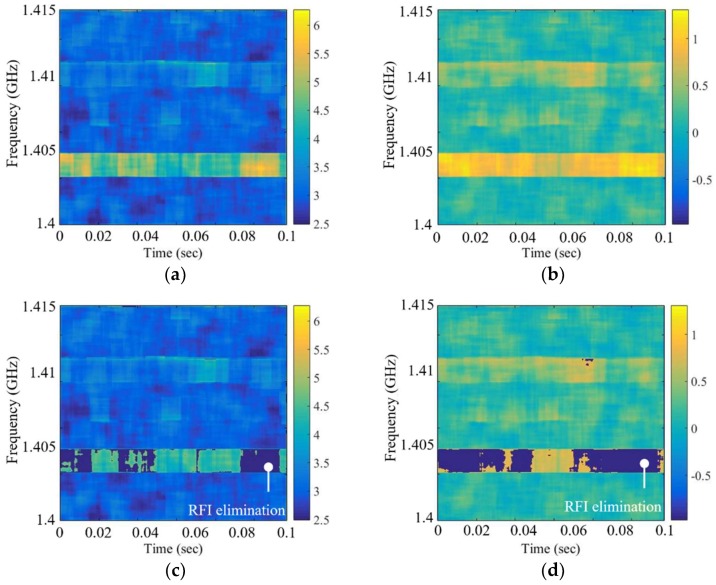
Example spectrograms applied to RFI-contaminated signals of approximately 25 K: (**a**) kurtosis spectrogram; (**b**) skewness spectrogram; (**c**) kurtosis spectrogram after thresholding; (**d**) skewness spectrogram after thresholding.

In Section 2, the skewness spectrogram is used to remove the RFI that can be detected. Similarly, RFI removal using kurtosis is also possible. Figure 12 shows the results of the skewness spectrogram and kurtosis spectrogram for a performance comparison. AM signal and pulsed signal with the same characteristics as in Section 4.2 are used under the same environmental conditions in Figure 6, and an RFI level of approximately 25 K is estimated to be the brightness temperature difference between the non-applied and applied RFI detection and mitigation algorithm. The kurtosis spectrogram can be obtained through the same procedure used to create the skewness spectrogram as shown in Figure 2. RFI contamination can be observed in the two spectrograms in Figure 12a,b; however, the removal results in Figure 12c,d are different when the threshold levels of the kurtosis spectrogram and skewness spectrogram are applied as 3 + 3*σ* and 0 + 3*σ* in those bins, respectively. Consequently, the skewness spectrogram shows that the performance of RFI elimination is better owing to the effect on the standard deviation and uncertainty of each spectrogram. Step 1 eliminates the detected RFI signals with not only a high level of the RFI signal but also a low level of the thermal signal, having the characteristics of an asymmetric distribution in the window. Specifically, the elimination of low-level signals is an important procedure to create a symmetric distribution from bins in the mean spectrogram reflected by RFI-flagged bins, because the bins on the left side of the distribution affect the computed kurtosis value in Step 2.

## 5. Conclusions

This paper describes a novel RFI detection and mitigation algorithm based on the spectrogram technique using the threshold level from statistical criteria. The proposed procedure is demonstrated with statistical approaches using skewness and kurtosis. In the experimental results, the maximum error of the retrieved brightness and RMSE are shown to be less than approximately 3 K and 1.7 K, respectively, with a window size of 100 × 100 from ten cases of RFI signals according to changes in the RFI level up to 100 K. The uncertainty of each bin in the skewness spectrogram can be reduced using a larger window size, and threshold level in the skewness spectrogram can be exactly determined. This affects the symmetric distribution from the left side of the distribution in Step 2, and the errors of the retrieved brightness temperature can be minimized. In summary, the error of the retrieved brightness temperature in the conventional method, which uses the threshold level with the mean value and standard deviation, is proportionally increased when RFI is spread over a wider range or stronger RFI signal. However, the proposed method can solve the problem of bias from zero in the skewness spectrogram instead of the mean value, and an appropriate value of the retrieved brightness temperature can be determined using kurtosis. Thus, the proposed method is an excellent candidate for RFI elimination compared with the conventional method. This experiment is considered using the analog RFI signal; however, the digital modulated signal, such as the phase-shift keying modulated signal, can be detected in the spectrogram from Step 1 [32], and this digital signal can be eliminated using kurtosis [33] and skewness [34] from Step 2. Thus, the digital modulated signal can be eliminated using the same method as the analog signal, as proposed in the experiment. Although the proposed algorithm was verified at the L-band, the microwave radiometer applications at higher frequencies can be applied for the purpose of measuring the earth’s environment such as sea surface temperature (SST), rain rates, ocean at C-band [7], and ocean wind at the Ku-band [9], and retrieving vegetation water content (VWC) at X-band [5]. This method is useful in the open ocean for satellites or on land in coastal areas with relatively low RFI effects; however, there may be the limitation of the RFI effect with very strong and wide-band RFI contamination, because the mean spectrogram and skewness spectrogram are covered by a wide-band RFI signal. Moreover, the ground-based radiometers observing scenarios with a higher range of brightness temperatures, such as warm spots, with respect to the environmental background can be difficult to apply to this algorithm because higher thermal emission signal can be misinterpreted as RFI signal [6]. An accurate brightness temperature value using the RFI detection and mitigation algorithm is required to obtain accurate information of the earth’s environment, and the proposed algorithm can be applied to the microwave band with additional performance evaluation across different frequencies. The proposed method will be applied to sea salinity [35] and soil moisture measurements along a coastline, and the performances in different RFI environments will be reported in the future. 

## Figures and Tables

**Table 1:** Instrument specifications of the developed L-band radiometer.

Index	Parameter
Frequency range	1.4–1.415 GHz
Antenna gain (dB)	10 Typ.
Radiometry sensitivity	<0.13 K
Radiometry stability	<0.2 K over a period of 1 h
Radiometry accuracy	<1 K over a period of 1 h
Integration time	1 s

**Table 2:** Maximum error of the retrieved brightness temperature (K) for different cases of RFI signals with the RFI level up to ~100 K.

RFI Signal Case	$\mathrm{Max}.\;\varepsilon_{T_{B}}\;(K)$ for Window Size			RMSE (K) for Window Size		
	50 × 50	75 × 75	100 × 100	50 × 50	75 × 75	100 × 100
Chirp signal	4.53	3.10	2.71	2.06	1.92	1.50
AM signal	3.69	3.07	2.89	1.55	1.51	1.31
Continuous signal	3.58	3.66	2.54	1.71	1.42	1.46
Pulsed signal	3.78	3.15	2.87	2.06	1.70	1.62
Continuous signal, pulsed signal	2.31	3.23	2.57	1.24	1.21	1.22
AM signal, pulsed signal	2.81	2.41	2.24	1.32	1.15	1.13
AM signal, continuous signal	3.95	2.43	2.76	1.50	1.49	1.52
Chirp signal, continuous signal	4.57	3.43	2.78	1.66	1.63	1.70
Chirp signal, pulsed signal	4.67	3.66	2.81	1.61	1.42	1.24
Chirp signal, AM signal, continuous signal	4.28	3.28	2.82	2.10	1.63	1.53

## References

[1] Oliva R., Daganzo-Eusebio E., Kerr Y., Mecklenburg S., Nieto S., Richaume P., Gruhier C. (2012). SMOS radio frequency interference scenario: Status and actions taken to improve the RFI environment in the 1400–1427-MHz passive band. IEEE Trans. Geosci. Remote Sens..

[2] Ruf C., Gross S., Misra S. (2006). RFI detection and mitigation for microwave radiometry with an agile digital detector. IEEE Trans. Geosci. Remote Sens..

[3] Querol J., Tarongí J., Forte G., Gómez J., Camps A. (2017). MER-ITXELL: The multifrequency experimental radiometer with interference tracking for experiments over land and littoral—Instrument description, calibration and performance. Sensors.

[4] Saleh K., Wigneron J., Waldteufel P., Rosnay P., Schwank M., Calvet J., Kerr Y. (2007). Estimates of surface soil moisture under grass covers using L-band radiometry. Remote Sens. Environ..

[5] Sawada Y., Tsutsui H., Koike T., Rasmy M., Seto R., Fujii H. (2016). A field verification of an algorithm for retrieving vegetation water content from passive microwave observations. IEEE Trans. Geosci. Remote Sens..

[6] Tasselli G., Alimenti F., Bonafoni S., Basili P., Roselli L. (2010). Fire detection by microwave radiometric sensors: Modeling a scenario in the presence of obstacles. IEEE Trans. Geosci. Remote Sens..

[7] Aksoy M. Evolution of the radio frequency interference environment faced by earth observing microwave radiometers in C and X bands over Europe. Proceedings of the 2018 IEEE International Geoscience and Remote Sensing Symposium.

[8] Bonafoni S., Alimenti F., Roselli L. (2018). An efficient gain estimation in the calibration of noise-adding total power radiometers for radiometric resolution improvement. IEEE Trans. Geosci. Remote Sens..

[9] Skou N., Lahtinen J. Measured performance of improved cross frequency algorithm for detection of RFI from DTV. Proceedings of the 2018 IEEE 15th specialist Meeting on Microwave Radiometry and Remote Sensing of the Environment.

[10] Alimenti F., Bonafoni S., Roselli L. (2017). A novel sensor based on a single-pixel microwave radiometer for warm object counting: Concept validation and IoT perspectives. Sensors.

[11] Roo R., Misra S., Ruf C. (2007). Sensitivity of the kurtosis statistic as a detector of pulsed sinusoidal RFI. IEEE Trans. Geosci. Remote Sens..

[12] Roo R., Misra S. (2008). A demonstration of the effects of digitization on the calculation of kurtosis for the detection of RFI in microwave radiometry. IEEE Trans. Geosci. Remote Sens..

[13] Guner B., Frankford M., Johnson J. (2009). A study of the Shapiro-Wilk test for the detection of pulsed sinusoidal radio frequency interference. IEEE Trans. Geosci. Remote Sens..

[14] Tarongi J., Camps A. (2009). Normality analysis for RFI detection in microwave radiometry. Remote Sens..

[15] Liang Z., Wei J., Zhao J., Liu H., Li B., Shen J., Zheng C. (2008). The Statistical meaning of kurtosis and its new application to identification of persons based on seismic signals. Sensors.

[16] Ruf C., Misra S., Gross S., Roo R. Detection of RFI by its amplitude probability distribution. Proceedings of the 2006 IEEE International Symposium on Geoscience and Remote Sensing.

[17] Roo R. A simplified calculation of the kurtosis for RFI detection. Proceedings of the 2008 IEEE International Geoscience and Remote Sensing Symposium.

[18] Kurtosis. https://en.wikipedia.org/wiki/Kurtosis.

[19] Ellingson S., Hampson G., Johnson J. Design of an L-band microwave radiometer with active mitigation of interference. Proceedings of the International Geoscience and Remote Sensing Symposium.

[20] Niamsuwan N., Johnson J., Ellingson S. (2005). Examination of a simple pulse blanking technique for RFI mitigation. Radio Sci..

[21] Guner B., Johnson J., Niamsuwan N. (2007). Time and frequency blanking for radio-frequency interference mitigation in microwave radiometry. IEEE Trans. Geosci. Remote Sens..

[22] Aksoy M., Johnson J., Misra S., Colliander A., O’Dwyer I. (2016). L-Band radio-frequency interference observations during the SMAP validation experiment 2012. IEEE Trans. Geosci. Remote Sens..

[23] Fanise P., Pardé M., Zribi M., Dechambre M., Caudoux C. (2011). Analysis of RFI identification and mitigation in CAROLS radiometer data using a hardware spectrum analyser. Sensors.

[24] Piepmeier J., Johnson J., Mohammed P., Bradley D., Ruf C., Aksoy M., Garcia R., Hudson D., Miles L., Wong M. (2014). Radio-frequency interference mitigation for the soil moisture active passive microwave radiometer. IEEE Trans. Geosci. Remote Sens..

[25] Tarongi J., Camps A. (2011). Radio frequency interference detection and mitigation algorithms based on spectrogram analysis. Algorithms.

[26] Skewness. https://en.wikipedia.org/wiki/Skewness.

[27] Ulaby F., Moore R., Fung A. (1986). Microwave Remote Sensing: Active and Passive, Volume I: Fundamentals and Radiometry.

[28] Frater R., Williams D. (1981). An active ‘cold’ noise source. IEEE Trans. Microw. Theory Tech..

[29] Schwank M., Wiesmann A., Werner C., Matzler C., Weber D., Murk A., Volksch I., Wegmuller U. (2009). ELBARA II, an L-band radiometer system for soil moisture research. Sensors.

[30] Goodberlet M., Mead J. (2006). Two-load radiometer precision and accuracy. IEEE Trans. Geosci. Remote Sens..

[31] Kenney J., Keeping E. (1951). Mathematics of Statistics.

[32] Sha’ameri A., Lynn T. Spectrogram time-frequency analysis and classification of digital modulation signals. Proceedings of the 2007 IEEE International Conference on Telecommunications and Malaysia International Conference on Communications.

[33] Gunther J., Moon T. (2009). Burst mode synchronization of QPSK on AWGN channels using kurtosis. IEEE Trans. Commun..

[34] Gupta A., Grossi M. (1986). Effects of nonzero-centroid and skewness of fading spectrum on incoherent FSK and DPSK error probabilities. IEEE Trans. Commun..

[35] Klein L., Swift C. (1977). An improved model for the dielectric constant of sea water at microwave frequencies. IEEE J. Ocean. Eng..

